# Relationship between Untreated Dental Caries and Weight and Height of 6- to 12-Year-Old Primary School Children in Bangladesh

**DOI:** 10.1155/2013/629675

**Published:** 2013-04-10

**Authors:** Masuma Pervin Mishu, Martin Hobdell, Mahfujul Haq Khan, Richard M. Hubbard, Wael Sabbah

**Affiliations:** ^1^Dhaka Dental College Hospital, Dhaka 1209, Bangladesh; ^2^University College London, Research Department of Epidemiology and Public Health, London WC1E 6BT, UK; ^3^Bangladesh Institute of Research and Rehabilitation in Diabetes, Endocrine and Metabolic Disorders (BIRDEM), Dhaka 1000, Bangladesh; ^4^Virginia Commonwealth University School of Medicine, Ashland, VA 23298-0565, USA; ^5^Oregon Health & Science University, School of Dentistry, 611 SW Campus Drive, Portland, OR 97239-3097, USA

## Abstract

*Background*. Children in low-income developing countries are likely to suffer from undergrowth. Dental caries is another common problem in these countries. *Aim*. To examine the association between untreated dental caries in primary and permanent teeth with age-adjusted height and weight among 6–12-year-old children in Bangladesh. *Design*. Social, behavioural, and clinical data were collected from 1699 children in nine different randomly selected primary schools in socially deprived areas of Bangladesh. The associations of age-adjusted weight and height and being underweight with dental caries were examined adjusting for sex, area of residence, socioeconomic position, skipping meals, tooth cleaning, and doctor visits. *Results*. 26% of the children were underweight and 55% had untreated dental caries. Children with at least one decayed tooth were significantly underweight with odds ratios 1.6 (95% CI 1.1, 2.3) and 1.5 (95% CI 1.1, 2.0) for 6–8-years and 9–12-year-old children, respectively, in the adjusted model. The number of decayed teeth was inversely and significantly associated with the standardized age-adjusted weight. *Conclusions*. The findings highlight the association between untreated dental caries and being underweight in primary school children in socially deprived areas in low-income developing countries and emphasize the need to integrate oral and general health policies with social policies.

## 1. Introduction

Dental decay is the most common childhood disease worldwide, and most of the decay remains untreated particularly in developing countries, [1, 2] thus affecting the growth and wellbeing of millions of children [3]. Dental caries in deciduous teeth among preschool children was found to be associated with being underweight and shorter [4, 5]. Furthermore, 12-year-old children with dental infections in permanent teeth had significantly below normal body mass index (BMI) [6]. Others have shown that treatment of dental caries in children was followed by weight gain [7, 8]. Different mechanisms have been postulated on the relationship between dental caries and child growth [9]. First, untreated caries could affect children's ability to eat it and, subsequently impairs adequate intake of nutrients [8, 10]. Infection from dental caries could also have impact on children growth [9, 11]. Furthermore, severe dental caries can affect quality of life including ability to sleep [12–14], which in turn impacts child growth [9]. On the other hand, others have suggested that the relationship between being underweight and dental caries is confounded by inadequate nutritional intake [15, 16] as poor nutrition can increase susceptibility to dental caries due to altered saliva composition and impaired secretion [17]. Earlier studies have also suggested a relationship between malnutrition, enamel hypoplasia, dental caries, and tooth exfoliation [18–21]. Others have also demonstrated that dental caries in primary dentition was associated with wasted and wasted and stunted children [21, 22].

Bangladesh is a low-income developing country with many children suffering from malnutrition and poor health, especially among the deprived sectors of the population [23]. Dental caries is also a common problem among children in Bangladesh [24], and the country has very limited facilities for dental treatment and a high population to dental provider ratio (100,000/2) [24]. This could possibly contribute to further deterioration of the nutritional status of the children in Bangladesh [9]. 

A couple of studies conducted on South Asian population have examined the association between dental caries and children growth [6, 25]. However, only one of them [6] has tested the hypothesis that dental caries has an impact on children growth using a relatively large sample and accounting for socioeconomic factors and personal cleaning habits. The objective of this study was to assess the association between age-adjusted weight and height with untreated dental caries in primary and permanent teeth among 6- to 12-year-old Bangladeshi children.

## 2. Material and Methods

The survey was conducted in 2007 in nine randomly selected primary schools across Bangladesh. Four slum urban areas, two suburban, and three rural sites were selected. The survey was approved by the Local School Committee, and consents were obtained from schools, students, and their parents. Data collection included dental clinical examination, anthropometric measurements, and the administration of a questionnaire.

Oral examinations were performed by two dentists who calibrated their assessment according to standard procedures described by World Health Organization [26]. The examinations were performed in the school-room with children in a seated position on a school chair; the examiner sat in front of them. Cotton pellets were used for drying the teeth, and natural day light and torch light were used for proper visibility. A CPI ball-ended probe and a lighted mouth mirror were used as examination tools to score caries according to standard procedures described by World Health Organization [26]. Initial caries lesions and early stages of cavitation where the ball-ended probe could not enter were not scored as caries.

All anthropometric measurements were performed according to standard guidelines [27]. The height of children, standing upright without shoes, was measured with a portable stadiometer to the nearest 0.5 cm. Weight was assessed with a portable electronic digital scale to the nearest 0.5 kg. No adjustments were made for clothing, but children were only lightly dressed. The measuring equipment was recalibrated daily. For the purpose of the analysis, two variables were created indicting weight-for-age *z*-score and height-for-age *z*-score. These variables are used by the WHO to estimate children growth [27, 28]. They indicate the number of standard deviation away from the mean (weight or height) for a specific age. An additional dichotomous variable was created indicating being underweight (weight-for-age *z*-score < −2) [28].

An interview questionnaire was administered which collected data on age, area of residence, socioeconomic position, and skipping meals, tooth cleaning frequency, and visits to a doctor. Age was categorized into two groups: 6–8 and 9–12, and the analysis was stratified by these two age groups. Area of residence was categorized into two groups: (1) urban and (2) rural and suburban. Socioeconomic position was based on a question pertaining to perception of socioeconomic status. This question was used to classify the children into two groups: (1) low and (2) low-middle socioeconomic position. The variable on skipping meals reflects the number of days per weeks when a child does not eat any meal. This variable was used as an extreme marker of poverty and was categorized as never and once or more. One oral health-related behaviour was included, namely, frequency of tooth cleaning with or without toothbrush (once a day versus more than once a day). The questionnaire also included a number of questions related to eating habits including consumption of sweetened snacks and beverages, meat, fish, fruits, and vegetables. Finally, doctors' visits was used a surrogate for general health conditions as it is very common in Bangladesh that people only visit a doctor when they are sick. The variable was used to indicate never visited a doctor in life or one or more visit.

## 3. Data Analysis

We first assessed the distribution of all variables used in the analysis for children with any decayed teeth and underweight children. The dichotomous association between being underweight and having at least one decayed tooth was examined for each age group. A series of linear regression models were constructed for each age group to assess the association between each of the two main outcomes (weight-for-age *z*-score and height-for-age *z*-score) and number of untreated caries adjusting for sex. Subsequently, the models were adjusted for area of residence, socioeconomic position, skipping meals. The final regression models were additionally adjusted for tooth cleaning and visit to a doctor.

Finally, logistic regression was used to assess the relationship between being underweight (weight-for-age *z*-scores < −2) and having at least one untreated decayed tooth, adjusting for sex, and subsequently to other covariates.

## 4. Results

A convenient sample of 1,720 children from nine schools was included in the survey. However, 1699 children were included in the analysis after excluding 21 who had incomplete data. The excluded children were not significantly different from those included in terms of weight, height, and dental caries. Overall 61% of the children had caries experience (DMFT/dmft > 0). The mean dmft and DMFT were 1.39 (95% CI 1.30, 1.49) and 0.35 (95% CI 0.30, 0.40), respectively. The majority of the affected teeth were untreated. Only 3.1% (mean: 0.01) and 13.7% (mean: 0.23) of the children had filled and extracted deciduous teeth, respectively. On the other hand, 3.1% of the children had extracted permanent teeth (mean: 0.4), and one permanent tooth had filling. Overall, 54.6% of the children had at least one untreated decayed deciduous or permanent tooth, and 26.4% were underweight. The prevalence of underweight was higher among boys, residents of urban areas, and those who skipped meals (Table 1). Children who had caries in one or more tooth (deciduous or permanent) were significantly more likely to be underweight in both age groups (Table 2).

In the linear regression model, for an additional decayed tooth, the weight-for-age *z*-score was significantly lower by 0.10 and 0.04 among 6–8-year- and 9–12-year-old children, respectively after adjusting of sex, socioeconomic position, skipping meals, tooth cleaning, and visits to a doctor. Similarly, for an additional decayed tooth, the height-for-age *z*-score was significantly lower by 0.11 and 0.05 among 6–8-year- and 9–12-year-old children, respectively (Table 3).

Finally, children with at least one decayed tooth were significantly more likely to be underweight with odds ratios 1.6 and 1.5 for age groups 6–8 years and 9–12 years, respectively, in the fully adjusted model (Table 4).

## 5. Discussion

This study examined the relationship of weight and height with untreated dental caries in 6–12-year-old children in Bangladesh. The results of the study demonstrated that children growth indicated by weight-for-age and height-for-age was inversely associated with dental caries. Earlier studies in developing countries reported similar negative correlations between anthropometric measures and the number of untreated carious surfaces and caries experience of the children [4, 5, 29].

Dental caries has a number of impacts on the daily activities of the children as it might impair their ability to eat or affect the quality of their sleep [4, 10, 14]. These impacts could be a possible explanation of the observed association in this study.

Many studies found that caries experience affects growth, specifically body weight and height of the children in an adverse manner [4, 10]. However, this association is confounded by other factors. Several environmental conditions may also explain social variations in oral health, which play important roles in the growth of children [15]. Dietary habits and conditions that are influenced by life style like dental caries, overweight, obesity, and malnutrition are shown to covary with socioeconomic status [30, 31]. Poor health in children, as in adults, is linked to lower social class and poor diet. Poor diet has a number of known adverse health effects including poor dental health, overweight, and underweight [32]. The high prevalence of dental caries is closely related to poverty and low socioeconomic status [33]. On the other hand, malnutrition of children is highly prevalent in low-income and middle-income countries, resulting in substantial increases in mortality and overall disease burden [34]. Bangladesh is one such country where two millions of children suffer from malnutrition [23, 35]. Growth is the best single measure for defining nutritional status and health of children as well as an indicator of quality of life [35]. Hence it is possible that the association between undergrowth and dental caries observed in this sample is comorbidity that can be attributed to the effect of deprivation.

In this study, the prevalence of undergrowth and dental caries was higher in urban slums where poor people live in crowded low quality housing conditions, with poor access to health services, insanitary facilities, unclean water, and no garbage collection and safe disposal. These living conditions contribute to ill health and malnutrition [35].

The cross-sectional design of this study is one of the limitations of this study as it does not allow establishment of the direction of the association. Furthermore, the study does not account for the time of tooth eruption, which could be delayed in undernourished children. The possible underestimation of the cariogenic exposure could have resulted in underestimation of the relationship between caries and children growth. Additionally, the children included in the study were all coming from deprived areas; hence, the variation in the impact of socioeconomic position on the children's growth was not fully captured.

This study demonstrated a relationship between untreated dental caries and undergrowth in a sample of Bangladeshi children. This relationship persisted even after adjustment for a number of socioeconomic and behavioural factors including dietary habits, such as consumption of sweetened snacks and drinks, fruits, vegetables, meats, and fish. The significance of the findings of this study is highlighting an additional health burden in low-income developing countries, namely, dental caries that could also have an impact on children's general health and quality of life [9]. Although evidence on the probable effect of untreated dental caries is increasing, but in developing countries, the impact of untreated dental caries on malnutrition and well-being is often ignored and underestimated. Furthermore, in low-income countries, oral health policies would usually be pushed to the bottom of the priorities or completely neglected considering the strained resources. Therefore, research findings that link oral health to general health problems specific to low-income developing countries present an opportunity to put oral health in the agenda of health care policies. The association of untreated dental caries with undergrowth among deprived children in Bangladesh observed in this study highlights the importance of integrating oral health policies with general health policies and with different social, political and environmental policies to address a cluster of health problems that share common determinants.

## 6. Conclusion

This study demonstrated a cross-sectional relationship between the untreated dental caries and being underweight and stunt in a sample of Bangladeshi children in poor areas. The association between dental caries, weight, and height was independent of selected socioeconomic and behavioural factors. The findings highlight the importance of oral health as an additional burden for children health in low-income countries. 

## Figures and Tables

**Table 1:** Description of study population (*n* = 1699).

Variables	Number	Within group percentage with caries	Within group percentage underweight
Age group			
6–8 years	754	60.0	29.2
9–12 years	945	50.4	24.2
Sex			
Boys	862	55.9	31.9
Girls	837	53.3	20.8
Area			
Rural and suburban	965	52.6	22.6
Urban	734	57.2	31.5
Socioeconomic position			
Lower	1069	53.6	26.1
Lower middle	630	56.4	27.0
Skipping meals in a week			
Never	1474	54.2	25.8
Once/more	225	57.8	30.7
Tooth cleaning			
Once/day	1161	54.2	26.9
Twice/day	538	55.7	25.6
Visit to a doctor			
Never	1214	52.1	26.1
Once/more	485	61.0	27.4

**Table 2:** Distribution of caries and underweight by age.

Age group	Any decayed teeth (*n*)	% of underweight children	Significance*
6–8 years	Yes (452)	33.4	<0.01
	No (302)	22.8	
9–12 years	Yes (476)	27.7	<0.05
	No (469)	20.7	

*P value of chi-square test for difference between caries groups.

**Table 3:** The association between weight-for-age *z*-score, height-for-age *z*-score, and the number of decayed teeth.

	Age group	Model	Regression coefficient	95% confidence interval	Significance
Weight-for-age *z*-score and untreated caries	6–8 years	First	−0.11	−0.16, −0.07	<0.001
		Second	−0.10	−0.15, −0.06	<0.001
		Third	−0.10	−0.15, −0.06	<0.001
	9–12 years	First	−0.04	−0.08, −0.00	<0.05
		Second	−0.03	−0.06, −0.01	<0.05
		Third	−0.04	−0.07, −0.01	<0.05

Height-for-age *z*-score and untreated caries	6–8 years	First	−0.13	−0.19, −0.07	<0.001
		Second	−0.10	−0.16, −0.05	<0.001
		Third	−0.11	−0.17, −0.05	<0.001
	9–12 years	First	−0.05	−0.10, −0.01	<0.05
		Second	−0.04	−0.09, 0.01	0.08
		Third	−0.05	−0.10, −0.01	<0.05

First model: adjusted for sex and Second model: adjusted for area description, socioeconomic class, and escape of meal per week. Third model: adjusted for tooth cleaning frequency and ever visited doctor.

**Table 4:** Odds ratio of association of underweight (weight-for-age <−2) with untreated caries adjusted for different variables.

Age group	Model	Odds ratio	95% confidence interval	*P* value
6–8 years	First	1.67	1.19, 2.33	<0.05
	Second	1.58	1.13, 2.23	<0.05
	Third	1.61	1.14, 2.27	<0.05

9–12 years	First	1.46	1.08, 1.98	<0.05
	Second	1.45	1.07, 1.96	<0.05
	Third	1.47	1.08, 2.00	<0.05

Weight-for-age *z*-score <−2 is under weight and ≥−2 is normal weight. Model 1: adjusted for sex. Model 2: adjusted for area description, socioeconomic class, and escape of meal per week. Model 3: adjusted for tooth cleaning frequency and ever visited doctor.
